# Acknowledgment to Reviewers of *Biomedicines* in 2021

**DOI:** 10.3390/biomedicines10020313

**Published:** 2022-01-28

**Authors:** Biomedicines Editorial Office

**Affiliations:** MDPI AG, St. Alban-Anlage 66, 4052 Basel, Switzerland

Rigorous peer-reviews are the basis of high-quality academic publishing. Thanks to the great efforts of our reviewers, *Biomedicines* was able to maintain its standards for the high quality of its published papers. Thanks to the contribution of our reviewers, in 2021, the median time to first decision was 17 days and the median time to publication was 36 days. The editors would like to extend their gratitude and recognition to the following reviewers for their precious time and dedication, regardless of whether the papers they reviewed were finally published:
A González-ArenasJosef IllekA. Pratap KumarJosé-Juan Pereyra-RodriguezAbby BenninghoffJoseph GeradtsAbdelrhman MenazeaJoshua SealyAbdullah Sami SaribaşJoshua TobinAbeer Al-GharaibehJosiah OchiengAbheepsa MishraJosip A. BorovacAbhigya MookherjeeJosko BozicAbhilash SamykuttyJoško OsredkarAbhinav VasudevanJozsef DudasAbhishek MishraJuan Antonio NavarroAbitha SukumaranJuan Bautista De SanctisAdam MieczkowskiJuan Carlos De VicenteAdele ChimentoJuan Carlos Rodríguez DíazAditya DandekarJuan Dominguez-RoblesAdrià ArboixJuan F. SantibañezAdriana AlbuJuan GambiniAdriana TrifanJuan Luis Rodríguez HermosaAdriano FriganovicJuan Miguel Martínez-GalianoAfshin BeheshtiJuan Mozas-MorenoAgata Faron-GóreckaJuan Pérez-FernándezAgata PucekJuan RoaAgata StanekJuan Rodríguez MansillaAglaia PappaJudit E. PongraczÁgnes KinyóJudith FischerAgnès LacroixJudith MichelsAgnese PoJuei-Tang ChengAgneta SimionescuJugal Kishore SahooAgnieszka A. KaczorJuilee RegeAgnieszka BlitekJulia CostaAgnieszka FilipekJulia SchumannAgnieszka KordekJulia StadlerAgnieszka KosowskaJulián AragonésAgnieszka MagryśJuliana M. IvanovaAgnieszka Małkowska-SzkutnikJuliana SaínAgnieszka MicekJuliann M. Di FioreAgnieszka Owczarczyk-SaczonekJulie Brogaard LarsenAgnieszka Piastowska-CiesielskaJulie Guillermet GuibertAgnieszka WaclawikJulie RageulAhmad Al-MrabehJulie WixeyAhmad JaliliJulien RossignolAhmed A. ElolimyJulien Saint-PolAhmed BakillahJulien Van GrevenyngheAhmed HasanJulio Francisco Jiménez-BonillaAhmet AyazJulita KulbackaAida PetcaJun FukiharaAiden EblimitJun ItamiAinara CanoJun WeiAinhoa AlberroJung Eun KimAjay DixitJung-hyun ParkAkhilesh RaiJung-Pan WangAkihiko MiyanagaJun-ichi HanaiAkihito TsubotaJunichi TakinoAkinari SawadaJun-Jen LiuAkira HigashimoriJunki SakataAlaa Abd-ElsayedJun-O JinAlaa AdawyJunseo OhAlak MannaJure KnezAlan Yueh-Luen LeeJürg HamacherAlba García-RodríguezJürg NüeschAlban GiraultJürgen PannekAlbert MoralesJürgen ThomaleAlberto MaccariJustin W. TaylorAlberto OuroJustyna DunajAlberto Rodríguez-MartínezJustyna KozłowskaAlberto SensiniJustyna Opydo-SzymaczekAlberto TommasiniJustyna StrycharzAlberto ZanellaJustyna ZińczukAlbino CarrizzoJyh-fei LiaoAldo ClericoJyh-Horng SheuAldona KasprzakK. GulyaAlejandro BugarinKa L. HongAlejandro Ibáñez-CostaKaare GautvikAlejandro Marin LopezKabirullah LutfyAlejandro Samhan-AriasKadir OzaltinAleksander SiniarskiKah-Chung LeongAleksandra KołotaKai KammersAleksandra KristoKai SonntagAleksandra Piechota-PolanczykKai YangAlenka Trampuš-BakijaKai YuAlessandra CostanzaKaixiong YeAlessandra GianoncelliKállai NikolettAlessandra QuartaKalpana RajaAlessandra ValerioKalyan SaginalaAlessandro AlaimoKamakoti BhatAlessandro AntonelliKamal JoshiAlessandro ArrigoKamalakannan VijayanAlessandro BonardiKamil GrubczakAlessandro ComandoneKamil KaminskiAlessandro E. P. VillaKamila Sroda-PomianekAlessandro FantiKandasamy SaravanakumarAlessandro FassinaKandhasamy SowndhararajanAlessandro GranitoKannan MuthukumarAlessandro GrinzatoKapil D. PatelAlessandro IeraciKarel SmetanaAlessandro MatteKaren AltAlessandro MaugeriKaren CrawfordAlessandro MaurielloKari KopraAlessandro StellaKarin Müller-DeckerAlessandro TrentiniKarina KwapiszewskaAlessio ReggioKarl BechterAlexander BerezinKarl G. StonecipherAlexander GasparskiKarla Mychellyne Costa OliveiraAlexander JaisKarmela BarisicAlexander LeeKarmele ValenciaAlexander M ShestopalovKarolina BoguszewskaAlexander RappKarolina M. StepienAlexander TH WuKarolina Mikulska-RuminskaAlexander ToliosKarsten GroteAlexandre EscargueilKarsten KnoblochAlexandre TrindadeKatalin BuriánAlexandru BurlacuKatarina LalicAlexei B. ChukhlovinKatarína ValachováAlexei N. SuminKatarina VukojevicAlexey G. MittenbergKatarzyna KaczyńskaAlexey KovalKatarzyna Knapczyk-StworaAlexey SarapultsevKatarzyna Komosinska-VassevAlexey SokolovKatarzyna Małolepsza-JarmołowskaAlexis JourdainKatarzyna Mleczko-SaneckaAlexis UlrichKatarzyna NeubauerAlfonso LagaresKatarzyna PiórkowskaAlfonso Martínez-NovaKatarzyna PiotrowskaAlfonso Olaya AbrilKatarzyna PlacekAlfredo Cabrera OreficeKatarzyna RoszekAlfredo CaturanoKatarzyna WalkiewiczAlfredomaria LuratiKatarzyna Wójcik-PszczołaAli BettaiebKaterina PsarraAli Ekrem YesilkanalKatharina RumpAli JazirehiKatherine MargulisAlice GualerziKathleen A. Welsh-BohmerAlice LuddiKathleen BörnerAlice OssoliKathleen HaleyAlice TurdoKathrin Bellmann-SickertAlice WitneyKathryn Y. BurgeAlicja KluczykKatia BarbaroAlin Laurenţiu TatuKatie SHEATSAlina AsandeiKatja FinkAlina KuryłowiczKatsuhiro ItoAline P. BeckerKatsuhito KinoAlisa SokolovskayaKatsuyoshi MatsunamiAlisha PrasadKausik ChakrabartiAllan R. MartinKavindra Kumar KesariAlok PaulKawaguchi NanakoAlvaro RendonKay Choong SeeAlyx TaylorKay Dietrich WagnerAmaju A. IkomiKazimierz KuliczkowskiAmal Kamal Abdel-AzizKazuhide OkudaAmalia StefaniuKazuhiko KuwaharaAmanda GendersKazuhiro ItoAmelia CimminoKazuhiro P. IzawaAmelia Maria GamanKazumoto MurataAmélie TrinquandKe WangAmie Peterson-HillerKei MaruyamaAmir Madany MamloukKeiichi IshiharaAmir Mohammad MalvandiKeith RochfortAmir RavandiKeittisak SuwanAmir ShemiraniKelly DomvriAmit KumarKelvin Ian AfrashtehfarAmna ParveenKen Kin Lam YungAmreen MughalKen KuduraAmro M. SolimanKenji HayashidaAmulya YaparlaKenji OheAmy MacNeillKenji OkiAna Bela Sarmento-RibeiroKenneth HettieAna Beltrán VelascoKenneth LundstromAna C. GonçalvesKenshirou ShiraishiAna Catarina TorresKenta KobayashiAna CerveraKenta MasuiAna CicvaricKerry LavenderAna Clara CristóvãoKerstin MenckAna CostaKerstin RohdeAna Cristina GonçalvesKeshari ThakaliAna DascaluKeshav Raj PaudelAna Fernández-SantanderKeunBaDa SonAna Margarida AbrantesKevin ChenAna Maria Santos-CoquillatKevin GlintonAna Marina FerreiraKévin MouzatAna MaroteKevin NashAna ReisKhashayar SakhaeeAna-Belen BlazquezKi Hyun BaeAnandharajan RathinasabapathyKi Hyun NamAnastasia BougeaKid TörnquistAnastasia De LucaKiichi HirotaAnastasios KoulaouzidisKijoon LeeAnastazia KeiKim TieuAnca BojanKimberley WangAnca DoceaKimitoshi SakamotoAnca Maria CimpeanKinga MusiałAnca Oana DoceaKippeum LeeAnca PanaitescuKiran SandhuAnca Roseanu ConstantinescuKirat K. ChandAnca-Maria CimpeanKirk BergstromAnca-Roxana PetroviciKirsten Haastert-TaliniAnders EsbergKirtikar ShuklaAnders Løbner-OlesenKishor Kumar SivarajAnders Palmstrøm JørgensenKishu RanjanAndleeb KhanKivilcim KilicAndrás BallaKiyoshi YaoedaAndrás BikovKlaudia SchossleitnerAndre CastroKlaus NeefAndré Da CostaKlaus-Dieter SchlüterAndre LechelKo FujimoriAndre WirriesKo OkumuraAndrea AgugliaKodeeswaran ParameshwaranAndrea AlexandreKohji FukunagaAndrea AmerioKoichi FujisawaAndrea ArmirottiKoichi OgamiAndrea BeccioliniKoichi ShinkaiAndrea ButeraKoichi TakimotoAndrea EwaldKoichi YamamotoAndrea FariniKoichiro OzawaAndrea FerrignoKok Siong ChenAndrea IorgaKoldo Garcia-EtxebarriaAndrea MarcinnòKonrad HuppiAndrea MartinelliKonstantin BelosludtsevAndrea MartiniKonstantin SlavinAndrea MoriondoKonstantina StathopoulouAndrea RonavariKonstantinos StergiosAndrea SambriKooi Yeong KhawAndrea TrevisanKornelia Kuźnik-TrochaAndrea Vukic DugacKoshiro NishimotoAndreas EbneterKostas A. PapavassiliouAndreas PetryKostas KasiotisAndreas PeyrlKosuke NishiAndree ZibertKota ArimaAndreea RatiuKotaro KigaAndreea-Teodora IacobKouichi MiuraAndrei LucaKrinio GiannikouAndreja Ambriovich RistovKrishna Prahlad MaremandaAndrés A. UrrutiaKristaps JaudzemsAndrés TittarelliKristen A. KrupaAndrew BeharryKristin Michaelsen-PreusseAndrew D. FrugéKristine M. KrajnakAndrew MillardKrisztina KovácsAndrew PoveyKrupal P. JethavaAndrey ElchaninovKrystyna OlczykAndrey I. GranovitchKrzysztof GornickiAndrey KozlovKrzysztof SztankeAndrey ReshetnikovKuldeep K. BansalAndrzej CzyrskiKun-Eek KilAndrzej TeisseyreKung-Woo NamAndy R. EugeneKunihisa MiwaAneta BalcerczykKuniyuki KanoAneta Cymbaluk-PloskaKuo-Ching MeiAneta Ostróżka-CieślikKuo-Hsuan ChangAneta WojdyłoKuo-Yen HuangAnette Gjörloff-WingrenKurt ZimmermanAnette WeyergangKwok Ho Christopher ChoyAngel De JuanasKyle Holmberg ViningÁngel SevillanoKyle PoulsenAngel Vizoso-VázquezKyoungmin ParkAngela Dalia RicciKyuho KangAngela LombardiKyung Pyo KangAngeliki AngelidiKyu-Sang ParkAngelo CarrettaKyuseok KimAngelo L. GaffoLadislav ŠtěpánekAnida Maria BabtanLampros PapadimitriouAniello MaieseLander Egana GorronoAniko GorbeLang ZhouAnirban ChakrabortyLarisa AnghelAnirban KarLarisa LitvinovaAnisoara CimpeanLars Ove BrandenburgAnita Lyssek-BorońLars P. KamolzAnja HornLars-Peter KamolzAnja LuxLasse LindahlAnja WeiseLaura AndolfiAnju KumariLaura BergantiniAnkana KakotiLaura BurattiAnkita PunethaLaura CianfrugliaA.N.M. Mamun-Or- RashidLaura Cristina RusuAnn SaadaLaura Elena SperlingAnna AngelousiLaura Francés-SorianoAnna AnnunziataLaura GrasaAnna BirkováLaura Inés Elvira-ToralesAnna Boroń-KaczmarskaLaura KennedyAnna CampanatiLaura LapagliaAnna CieślińskaLaura LocatelliAnna D. SerefkoLaura MarchettiAnna Herman-AntosiewiczLaura MaziluAnna Kablak-ZiembickaLaura Mora-LópezAnna La SalviaLaura MusazziAnna LewinskaLauréline RogerAnna LopataLaurent DelpyAnna M. OsyczkaLaurent L'hommeAnna Makuch-KockaLaurentiu PopAnna Maria BassiLauro Velazquez-SalinasAnna Maria MalfitanoLavinia RaimondiAnna Martina BattagliaLawrence E. GoldfingerAnna Merwid-LądLeah StiemsmaAnna MrzljakLech B. DobrzańskiAnna N. BerlinaLechuang ChenAnna NowakLee Ann Laurent-ApplegateAnna OniszczukLeela Raghava Jaidev ChakkaAnna Palko-LabuzLeena LatonenAnna PasettoLeena P. BharathAnna PilutinLeif BülowAnna RamunnoLeigh PlantAnna RoschkeLeigh WardAnna SabateLen LuytAnna SieroslawskaLenka MichalcovaAnna Stanislawska-SachadynLeonardo AlbarracínAnna Tankiewicz-KwedloLeonardo ErminiAnna WilkaniecLeonardo Saúl Lino-SilvaAnna Winiarska-MieczanLeonid Pavlovich ChurilovAnnafranca CavaliereLeonidas DuntasAnna-Liisa NieminenLesa A. BegleyAnnalisa ChiavaroliLeszek KrólickiAnnalisa CiabattiniLetizia MezzasomaAnnalisa De SilvestriLeu-Wei LoAnnalisa MasiLew Lee ChingAnnamaria MassaLeyre ZubiriAnnie KathuriaLi OuAnnika FendlerLia GinaldiAnnika ÖhrfeltLia H. CampbellAnoop RawatLi-Ang LeeAnouk W. VaesLiang WangAntal NógrádiLiang XuAnthony SadlerLiborija Lugović-MihićAntje NeebLidia Hanna MarkiewiczAnton V. DolzhenkoLidia MartínezAntonella BisioLígia C. Gomes-da-SilvaAntonella CamaioniLihua ChenAntonella FrassanitoLijuan FengAntonella MottaLilach SoreqAntonella OliveroLili YangAntonella RomaniniLiliana S. MendonçaAntonella RosiLilianne BeolaAntonella ZannettiLilong GuoAntonia FeolaLin ZhuAntonia LopresideLinbo ZhaoAntonin KrajinaLinda MontemiglioAntonio AndresLindsey Van HauteAntonio AversaLingling MiaoAntonio BiondiLingxiao XieAntonio BrucatoLionel HebbardAntonio C.P. WongLitia Alves De CarvalhoAntonio González-BulnesLiudmila V. SpirinaAntonio Henrique MartinsLivio LuongoAntonio LucacchiniLoka PenkeAntonio ManciniLong Y ChiangAntonio ManentiLóránd KissAntonio Mario ScanuLoredana MarcuAntonio Martinez-LopezLoredana UrsoAntonio MichelucciLorena Del Pozo-AceboAntónio Miguel MorgadoLorenz H. LehmannAntonio OrlacchioLorenzo CarnevaleAntonio Q. TonioloLorenzo GenitoriAntonio SerranoLorenzo OnoratoAntonio Simone LaganàLorne BabiukAntonios MakridisLory Saveria CrocèAnurag SharmaLouis PenningAnushree AcharyaLouise O'HareAránzazu MedieroLourdes MounienArati SridharanLourdes Rivas TorcatesAravind KshatriLubomir SkladanyAria BaniahmadĽubomíra GrešákováAriadni SpyroglouLuc BoussetAriela BurgLuca BurroniArif LuqmanLuca De SimoneAris TsalouchosLuca Di GianfrancescoAristeidis PapagiannopoulosLuca FalzoneArlyng GonzalezLuca GallelliArmin MooranianLuca GiacomelliArmond DaciLuca MezzettoArnika Kathleen WagnerLuca PangrazziArnold GrünwellerLuca SimulaArnoldo RiquelmeLucia BrodosiÁrpád Ferenc KovácsLucia CarulliArshi KhanamLucia MorbidelliArtem BlagodatskiLucia ScisciolaArtemissia-Phoebe NifliLucia StančiakováArtur PałaszLuciana GiardinoArtur TurekLucija Peterlin MašičArturo CesaroLucija Virovic-JukicArup ChakrabortyLucio Diaz-FloresAsaf AchironLudek SlavikAshit RaoLudmila KazdovaAshley P. NgLudmila V. PuchkovaAshok MandalaLudovic PelosiAshu JohriLudwig WilkensAsmerom SengalLuigi Di FilippoAssunta PozzuoliLuigi GianturcoAthanasia PrintzaLuigi PetramalaAthanasios SaratziotisLuigi RussoAtsushi KoikeLuis Antonio Marinas-PardoAttila FrigyLuis Fernandez De CastroAttila KeresztesLuis GandíaAtul SharmaLuis García-FernándezAudrey VarinLuis GoyaAurelian-Sorin PașcaLuis M. Alonso-ColmenarAurora ArghirLuís M. FélixAurore FraixLuis Peña QuintanaAustin B BigleyLuís Pinto Da SilvaAvijit PramanikLuis RodrigoAvishek MitraLuisa AgnelloAviv ShaishLuiz Gustavo ChuffaAyumi SugiuraLukas LacinaAzzurra StefanucciŁukasz A. PoniatowskiBabu V. SajeshLukasz KurykBach Quang LeŁukasz MałekBachir BenarbaŁukasz SobottaBaiba SvalbeŁukasz SzeleszczukBaicus AndaLuke J. NeyBal Krishna ChaubeLulu HuangBalaji KrishnamacharyLuminita PaduraruBalamuralikrishnan BalasubramanianLutz KonradBalázs GasznerLynne A. FieberBalázs MayerLyuba Dineva MitevaBalik DzhambazovM. Azizur RahmanBálint NagyM. Dolores MolinaBalupillai AgilanM. Rashed KhanBaptiste LamarthéeM. Sheikh MohamedBarbara De AngelisMaciej Iek SkrzypczakBarbara DziedzicMaciej JarzebskiBarbara DziegielewskaMaciej MisiolekBarbara LiederMaciej SkrzypczakBarbara LipińskaMaciej ZielińskiBárbara Mendes-PinheiroMaddalena Di SanzoBarbara RuaroMadelyn GillentineBarbara Ruszkowska-CiastekMadhu OusephBarbara ToffoliMadhumita ChatterjeeBartlomiej BarczynskiMagda R. HamczykBartosz MiziołekMagda SpellaBartosz PulaMagdalena BaymakovaBartosz TrzaskowskiMagdalena Chottova DvorakovaBeata Butruk-RaszejaMagdalena GrillBeata BystrowskaMagdalena Jeszka-SkowronBeata Kasztelan-SzczerbinskaMagdalena Krajewska-WłodarczykBeata M. Gruber–BzuraMagdalena LepickaBeata Morak-MłodawskaMagdalena Martínez-CañameroBeata NowakMagdalena SkarzynskaBeatriz Martín-AntonioMagdolna NagyBee K TanMagrassi LorenzoBehnam RashidiehMahdi GarelnabiBelén Begines RuizMahdi ShahmoradiBenedict SeoMahmood MozaffariBeniamin Oskar GrabarekMahmoud Al-KhrasaniBenjamin BitlerMahoney John MatthewBenjamin H. DurhamMaike BuschBenjamin R. CaroneMaitane AsensioBenoit MichotMaja MatulicBenoit MiottoMaja MatulićBernadette M.M. ZwaansMajed OdehBernardete F. MeloMajid MomenyBernhard BiersackMakoto EndoBernhard RyffelMakoto HashimotoBernhard ZelgerMakoto KobayashiBerta CasarMakoto NaoiBettina BlaumeiserMakoto TsunodaBhagyaraj EllaMalcolm McConvilleBharani Krishna Mynampati ArunadithyaMałgorzata Anna MarćBiao ZengMałgorzata DołowyBijay DhungelMałgorzata GrembeckaBiliana NikolovaMalgorzata KajtaBimal KoiralaMalgorzata KlocBishnubrata PatraMałgorzata LenartowiczBishweshwar PantMałgorzata Polz-DacewiczBiswarup SahaMalgorzata Sztiller-SikorskaBjörn TampeMallesh KurakulaBjörn WindshügelMan TongBo WangManabu NiimiBoas PuckerManash K. PaulBoaz BarakManel Ben HammoudaBogdan CatargiManijeh PasdarBogdan Silviu UngureanuManish JainBogdan SmykManish KumarBoleslaw KarwowskiManoj GovindarajuluBong-Hyeon KyeManoj MishraBongju KimMansoureh TatariBonsu KuManuel Jiménez-TenorioBoris SlobodinManuel Pardo-RiosBorja Herrero De La ParteManuela CabiatiBoryana PetrovaManuela CervelliBoyong KimManuela SabatinoBozena KaminskaManuela ViolaBożena Romanowska-DixonMao-Meng TiaoBradley N. SmithMar CarriónBram SlütterMara MazzoniBrandon S. PruettMarat EzhovBrent PageMarc HilhorstBrian D. AdamsMarc MauryBrian M. WardMarc ParmentierBrigitta ButtariMarc PoirotBrigitte KerfélecMarc YesteBrigitte MarianMarc-André LécuyerBruno FiondaMarcello MocciaBryan D. CrawfordMarcin FeldoBryan J. MathisMarcin HeljakBrzozka ZbigniewMarcin JozwikBulat RamazanovMarcin NicosBurkhard PoeggelerMarcin SamiecByeong-Wook SongMarcin WłodarczykByron BaronMarco Bauzá ThorbrüggeByung Soo KimMarco BortolusCălin Bogdan ChibeleanMarco CascellaCallista MulderMarco FerrariCalogero DiBellaMarco G. AlvesCamila D. Madeira CamposMarco GovoniCamille MaloufMarco GuerriniCan CuiMarco LanzillottaCandan HizelMarco MascittiCankut ÇubukMarco RossiCao HuangMarco TatulloCarina MihaiMarcos Arturo Martínez-BanaclochaCarl D. BortnerMarcus BahraCarl Friedrich ClassenMarcus GrimmCarl MousleyMarcus SchmidtCarla CruzMarek KonopCarla LopesMarek KrzystanekCarla PeregoMarek LubośnyCarlo BarausseMarek MuriasCarlo BarbagalloMargaret SchnitzlerCarlo CastellanoMargarita A. SazonovaCarlo GalliMargarita Jimenez-PalomaresCarlo LajoloMargarita ParraCarlos Del Fresno SánchezMargarita TenopoulouCarlos FarinhaMargherita G. De BiasiCarlos Fernando MourãoMargherita MorpurgoCarlos Guerrero-MosqueraMaria A. BonifacioCarlos OpazoMaria A. VulfCarlos PlanchaMaria AndreassiCarlos SequeiraMaría Angeles PérezCarmela ParentiMaria Angeles RojoCarmelo BiondoMaria Antonia FrassanitoCarmen GaidauMaria BabakCarmen HerenciaMaria BloksgaardCarmen RamírezMaria ContaldoCarmen RinaldiMaria D. Giron-GonzalezCarmine FinelliMaria Dolores Vázquez-CarreteroCarmine OstacoloMaria Elisabete C.D. Real OliveiraCarol Yen Chin LinMaría EstebanCarole HéniqueMaria Gabriella GabrielliCarolina De La TorreMaria GazouliCarrie M. ElksMaria Giulia BattelliCarsten DrepperMaria Grazia BottoneCassie S. MitchellMaria Grazia Di CertoCatalin TiliscanMaria Grazia MolaCatalina Sanz-LozanoMaria Helena CasimiroCatalina Vallejo-GiraldoMaria HyttiCatarina MirandaMaria IskraCaterina Maria GambinoMaría Jesús Alvarez CuberoCaterina PalleriaMaría Jesús Núñez-IglesiasCaterina Peraldo-NeiaMaría Jesús Rodríguez-YoldiCatherine CoiraultMaria João MenesesCatherine DuezMaría José García-BarradoCatherine StefanatoMaria Laura IddaCathleen R. CarlinMaria LeeCátia I.C. EstevesMaria Llanos CasanovaCécile ClercxMaría M. EscribeseCecile VoissetMaria MoschoviCecilia B ChighizolaMaria N. BalatskayaCecilia NalliMaria PantelidouCedric HermansMaria Paola ParonettoCedric NeveuMaria PapadakiCelia Andreu-SánchezMaria Pia FerrazCelia CruzMaria PratCéline DevroyeMaria RodriguezCéline LoinardMaria Serena FabbriniCésar Calvo-LoboMaría Teresa Agulló-OrtuñoCezary KowalewskiMaria Teresa GentileChang Geen-DongMaría U. MorenoChang-Liang ChenMaria VinciChang-Min LeeMariamena ArbitrioChangmo HwangMariana SantosChang-Shen LinMariana TudoranChao-Wen LinMariana VarnaCharalampos SiristatidisMariangela De RobertisCharalampos ThomasMariangela GarofaloCharat ThongprayoonMariann HarangiCharis LiapiMarianna BarbalinardoCharles EberhartMarianne BrodmannCharlotte GenestetMarianne VozCharu KothariMarianneza ChatzipetrouChe-Hsin LeeMariastefania AnticaChen Huei LeoMarie BillaudChenglei LiMarie C BénéCheng-Maw HoMarie Nicod-LalondeCheng-Shun LuMarielle SaclierCheng-Wei LiuMarie-Pierre GolinelliCheng-Yang HuangMarie-Pierre GratacapCheng-Ying ChuMarie-Thérèse Dimanche-BoitrelChen-Hua LiuMarietta HerrmannChenyi PanMarija JozanovićCheol HwangboMarika CordaroCheol-Su KimMarika TardellaCher-Rin ChongMarilena CeccarelliCheuk Lun LiuMarin MicutzChi Chien LinMarina FialloChi Keung LamMarina KhodanovichChia-Chen ChangMarina OteleaChia-Jung LiMarina PiscopoChian Ju JongMarina SalaChianju JongMarine TheretChiara CaselliMarino VilovićChiara Da PieveMario AbateChiara GabelliniMario De FeliceChiara VillaMarion CurtisChia-Yih WangMarios G. KrokidisChia-Yuan ChenMariusz HartmanChi-Chen LinMarjeta TerčeljChien-Feng LiMarjorie PionChien-Ming ChaoMark A. PotterChien-Tai HongMark ClemensChi-Fen ChangMark S. KindyChih Hung LoMarko JukičChih-Chia HuangMarkus HenningssonChih-Li LinMarkus VeltenChihyang WangMarlena ZyśkChii-Wann LinMarta Andres-MachChikafumi ChibaMarta FichnaChi-Li ChungMarta GalloChi-Ming WongMarta GarcíaChing-Jin ChangMarta Gil BarbaChingyu LinMarta KepinskaChi-Ping LiMarta LualdiChi-Wen LuoMarta MonzónChi-Ying F. HuangMarta PutrinšCho Yeow KohMarta R. RomeroCho-Hao LeeMarta RieraChoyi ChenMarta RybskaChris A. FlaskMarta ToczekChrista BuechlerMarta TomaszkiewiczChristel MarquetteMarta WoźniakChristel NeutMarta Z. PaciaChristelle MonvilleMartin ChanChristelle PougetMartin G. O'TooleChristian DaniMartin HundChristian IneichenMartin MichaelisChristian KuttnerMartin PootChristian MüllerMartin PrusinkiewiczChristian OpländerMartin TeraaChristian PetersMartin Villalba GonzalezChristian ReynoldsMartin VlkChristian WirajaMartin WawruchChristina KaravasiliMartina BonifaziChristina LeysonMartine DesroisChristine CheungMartins FerreiraChristine E. EngelandMartyna Krzykawska-SerdaChristine EngelandMary BeilbyChristoph BiehlMarzena LaskowskaChristoph GeislerMaša SinreihChristoph ReinhardtMasaaki KonishiChristophe A. MarquetteMasabumi ShibuyaChristophe CoudretMasahiro ImamuraChristophe JacobMasahiro MaruyamaChristopher J WorsnopMasakazu SugishimaChristopher KobierzyckiMasaki MiyasakaChristopher L. CoeMasakiyo SasaharaChristos PapaneophytouMasakiyo YatomiChrysovalantis KorfitisMasanori MurakamiChuanbin YangMasaru TanakaChuen-Mao YangMasashi KanaiChuhee LeeMasashi MukohdaChul-Su YangMasashi TanakaChunchia ChengMasato YanoChun-Chih TsengMasatsune OguraChung-Jung LiuMasayo TakahashiChun-Jung ChenMasmudur M. RahmanChun-Lin ChenMassimiliano EspositoChunmin C. LoMassimiliano PetriniChun-Ru ChienMassimo CarossaChunye ZhangMassimo ConeseChunyi WuMassimo LibraCinzia RotondoMáté VargaCiprian RezusMatea Nikolac PerkovicCiro CelsaMateusz GliwińskiCiro Di NunzioMateusz PierógCiro ManzoMathias MontenarhClaire JosseMatilde Yung FolloClara BalsanoMatteo BaucknehtClarissa BerardoMatteo ChiappediClaudia AltomareMatteo Cotta RamusinoClaudia CrociniMatteo FermiClaudia CrosioMatteo GiovarelliClaudia CurciMatthew BeneschClaudia De VitisMatthew BottomleyClaudia PisanuMatthew C. DavisClaudia Rangel-BarajasMatthew HarriesClaudia RiccardiMatthew PanClaudia ValverdeMatthew T. WolfClaudio FarinaMatthias David ErlacherClaudio LuchiniMatthias DerwallClaudio RossiMatthias Hardtke-WolenskiClaudio UcciferriMatthias MeinhardtClaudiu N. LunguMatthias NeesClotilde CostaMatthias W. LaschkeClyde F. PhelixMatthieu RuizCodruta SoicaMatti JauhiainenColin McInnesMattia ChiesaColleen CronigerMattia Di PaoloConcetta Elisa OnestiMattia Falchetto OstiConcetta SaponaroMaureen H.V. FernandesCongBao KangMaurizio ForteCongshan SunMauro ManiscalcoConstantino MartínezMauro MontalbanoConxita Jacobs-CacháMax NibertCornelius FaberMax PiffouxCorrado AngeliniMaximilian Christian SchieleinCorrado PelaiaMaximilian SeidlCorrado RomanoMay GriffithCosimo BruniMaya DymovaCosimo CumboMayumi KomineCostantino BalestraMayur ChoudharyCraig J. GoergenMd Ashik UllahCraig M. GoodwinMd. Habibur RahmanCristian FurauMd. JakariaCristian MunteanuMd Jamal UddinCristian StătescuMd. Nazmul IslamCristian TuratoMedha PriyadarshiniCristiane Koga-ItoMehmet M AltintasCristiano AmarelliMei-hwa LeeCristina AntinozziMeiyun FanCristina CarvalhoMelanie Haffner-LuntzerCristina ChircovMelchor Sanchez-MartinezCristina MarchiniMelike FirlakCristina Martin-SabrosoMelissa EdwardsCristina NicaMelissa R. AndrewsCristina NombelaMelissa SantiCristina PopMengyu WangCristina Rosell-ValleMeong Cheol ShinCristina SegoviaMercedes CamachoCristina SoléMercedes CostellCristoforo ComiMercedes FernandezCristoforo PomaraMercedes Garcia-GilCsaba HetényiMetello InnocentiCSandra SobocanecMetin UzCsilla Özvegy-LaczkaMetodi D. MetodievCurtis FrenchMichael A. JacksonDae-Kwang KimMichael AlkanDagmar HorákováMichael D. SpartalisDaiki MuraseMichael HofmanDaishi YamakawaMichael Jordan RowleyDaisuke InoueMichael RutzlerDaisuke KaidaMichael SchaeferDaiva BironaitéMichael V. UgrumovDalius JatužisMichael Van DykeDamian Jozef FlisMichaela FiliouDamiano FantiniMichał BiernackiDamien N. StringerMichał BijakDamir SapunarMichał Błazej PonczekDamu TangMichał ChmielewskiDan SongMichal DoušaDan ZhaoMichał GinsztDana Maria CopoloviciMichal J. DabrowskiDanail PavlovMichal JurasekDancho DanalevMichal JurášekDaniel G. BichetMichal KlichowskiDaniel HierMichal LetekDaniel Lopez-MaloMichał OczkowskiDaniel P. PotaczekMichal OrdakDaniel ReuterMichał StrzeleckiDaniel Sobrido-CameánMichał ZarobkiewiczDániel TöröcsikMichele D'AngeloDaniel TurudićMichele FerraraDaniel W. NixonMichele GhidiniDaniela BraconiMichele GraciottiDaniela GabbiaMichele GregórioDaniela MessineoMichele MadiganDaniela MiricescuMichele MalaguarneraDaniela R. RecchiaMichele NavarraDaniela Valeria MinieroMichele RedellDaniela-Saveta PopaMichèle SalmainDaniele MasaroneMichelle MitchenerDaniele PironiMieczyslaw PokorskiDaniele PiscitelliMiguel A OrtegaDaniele VergaraMiguel A. OrtegaDanielle A. GuarracinoMiguel BurgosDany Munoz-PintoMiguel CarracedoDarin ZertiMiguel HuesoDario Di NardoMiguel VelazquezDarya NovopashinaMiguel Vicente-ManzanaresDaumantas MatulisMihaela R. PopescuDavid Baglietto-VargasMihai NitulescuDavid Bar-OrMihai Stefan MuresanDavid BurnsMihaiela Cornea-CipciganDavid ConversiMihaly DernovicsDavid CoquerelMiika MartikainenDavid FitzgeraldMika HoriDavid LeitschMikhail DubininDavid M. DiamondMikio HayashiDavid MontsMilad PourbaghiDavid ObesoMilan JakubekDavid OrnellesMilena G. GeorgievaDavid Ramiro-CortijoMilena GeorgievaDavid RamsdenMilena ŚciskalskaDávid SzatmáriMilki RahatDavide BarrecaMiloš I. DjuranDawid JanasMilos PjanicDawid PrzystupskiMin ParkDaxesh P. PatelMin Young UmDay-Shin HsuMinchan GilDean G. PhelanMing Feng LiaoDean SmithMingchuan ZhouDebbie C. CransMinglei GuoDebopam GhoshMingming TongDéborah Tribouillard-TanvierMingsheng ChenDeclan P. McKernanMing-Tzu TsaiDeepak SinghMing-Yow HungDejan GeorgievMin-Jung KangDejan JakimovskiMinxiang ZengDejan NikolicMinzhong YuDe-Li ShiMircea Ioan PopaDemetrios ArvanitisMireia MallandrichDenis BourgeoisMirela Baus LončarDenis BuxtonMirella GiovarelliDenis KainovMiriam HickeyDenis NazarovMiriam ZacchiaDenisa MarginaMiriana D’AlessandroDer-Yuan ChenMirjam SchuchardtDessie Salilew-WondimMirjana KesslerDeven PatelMiroslaw JanowskiDhananjaya KulkarniMiroslawa CichorekDhanush HaspulaMitsuhiro TakenoDi Girolamo Filippo GiorgioMitsuru FutakuchiDi Miceli MathieuMitsuru MizunoDi WuMitsuteru AkahoshiDiaa AhmedienModesto De CandiaDiana CostaMohamad AlamehDiana GuimarãesMohamad AssiDiana MartinsMohamed A. HassanDiana SilvaMohamed Ali Abdel-RahmanDiego Francesco CalvisiMohamed El-ShazlyDiego HaroMohamed MahdiDiego RaimondoMohammad Asif SherwaniDiego VelascoMohammad Hassan BaigDieter SchmollMohammed A. RohaimDietmar EnkoMOHAMMED BARIGOUDietmar FuchsMohanraj SadasivamDik-Lung MaMoises Armides Franco-MolinaDiletta Francesca SquarzantiMoises Di SanteDilpreet BuxiMojca KržanDimitri PoddigheMokarram M. HossainDimitrios KontogiannatosMona FothDinara SobolaMona K. JomaaDion BrocksMona ShehataDirk HecklMonica GelzoDirk MontagMónica MartinsDirk WohlleberMonika FurkoDisha SharmaMonika Klinkhammer-SchalkeDivya MurthyMonika SzeligaDivya RamchandaniMonokesh Kumer SenDmitri V. NikitinMontecucco FabrizioDmitriy V MazurovMootaz SalmanDmitry V. ChistyakovMoritake IguchiDobrila NesicMorten LadekarlDolzhikova InnaMostafa RatebDomenico NuzzoMoumita DattaDomenico PalumboMudit MishraDominic Paquin-ProulxMuhammad Imran RahimDominik DłuskiMuhammad Saad KhanDominik PoradowskiMuh-Shi LinDominik S. SkibaMulazim HussainDominika GłąbskaMumtaz AnwarDominika ZającMumtaz V. RojianiDominique P. GermainMunari FabioDomitilla MandatoriMunekazu YamakuchiDonato CalabriaMurali MamidiDonato Di VenereMurugan SubbiahDong ChenMurugesan VelayuthamDong Yun LeeMuthukumar BalasubramaniamDong-Hoon JinMuzamil Yaqub WantDongyun ZhangMyeounghoon ChaDoreen LauNabil RashdanDorota Tichaczek-GoskaNadia CalabrisoDorota WrześniokNadia JahroudiDorothea K. ThompsonNadia Maria SposiDoyeon KimNaeem KhanDragana NikitovicNagalingam RajakumarDragana Vasic AnicijevicNagaraja S. BalakathiresanDragos Catalin JianuNai-Jen ChangDragos MedianNanako KawaguchiDrucilla J. RobertsNaoaki SakataDuaa DakhlallahNaoki KawagishiDuarte PignatelliNaoomi TominagaDubravka SvobNaoro TanakaDuc-Thang VoNaoyuki KataokaDulskas AudriusNarendar DudhipalaDya Fita DibweNatalia A. KuzminaDylan Jack RichardsNatalia BabyshkinaDzung H. DinhNatalia Garcia-GiraltEberhard GrambowNatalia GrubaEdith HoferNatalia GuevaraEdmund CarvalhoNatalia KordulewskaEdmundo Rosales-MayorNatalia LinkovaEdoardo RosatoNatalia LisiakEdoardo StaderiniNatalia MadetkoEfrat Shavit-SteinNatalia ManousiEgizia FalistoccoNatália MartinsEhsan AhmadpourNatalia MiękusEiji KawamotoNatalia N. NinkinaEijiro JimiNatalia N. ShestakovaEileen ShoreNatalia RakislovaEkaterina A. LitvinovaNatalia V. BelosludtsevaEkaterini ChatzakiNatalia V. GulyaevaElba MaurizNatalia VydraEleanor LedererNatalia Wawrusiewicz-KurylonekEleftheria GalatouNatalie A. GudeElena AndreevaNataliya KolosovaElena AnghileriNatella EnukashvilyElena BoggioNathalie GuriecElena ConteNathan K. EvansonElena D. BazhanovaNathan L. AbsalomElena DinteNathan PennockElena EfremenkoNeeraj ShahElena FicoNeha NandaElena GargaunNehal ElsherbinyElena GubarevaNeil CarletonElena Ivanovna ShramovaNela PivacElena KosenkoNeus CarbóElena LeychenkoNguan Soon TanElena Martín-GarcíaNiall MurphyElena ObradorNicholas Agung KurniawanElena PiccininNicholas G KounisElena PopugaevaNicola FerriElena SergeevaNicola FuscoElena SorokinaNicola ZampieriElena V. ProskurninaNicolae BacalbașaElena V. TchetinaNicolae GicaElena VeronesiNicolas DemaurexElena VismaraNicolas L'HeureuxEleni AnastasiadouNicolas SadgroveEleni MavrogonatouNicolas SantiquetEleonora Da PozzoNicolas TaulierEleonora MarsichNicole BechmannEleonora TimperiNicole N. HaeseEleonore FröhlichNicolòmaria BuffiEli BoritzNien Mu ChiuEli D EhrenpreisNihar KinarivalaElikanah Olusayo AdegokeNiharika DuggalEline MeulenbergNikolaos AntonakopoulosEline Pecho-VrieselingNikolaos N. LourosElisa BelluzziNikolay MehterovElisa CairrãoNikolina Maček HrvatElisa GiovannettiNileshkumar DubeyElisa MartellaNiraj NepalElisa NavarroNobuaki MichihataElisa PanzariniNobuaki OkumuraElisabeth Karin Maria MackNobuhito GodaElisabetta DonzelliNobuyuki ShimozawaElisabetta LiveraniNoemí EiróElisabetta RazzuoliNoor KazimElisavet StavropoulouNor Eddine SounniElise FouquerelNora Jean NealonEliyahu DremencovNorbert KissElizabeth Helen KempNorbert NassElizabeth ThomasNorbert NighoghossianElke Pogge Von StrandmannNorbert StefanEllas SpyratouNorihiko NaritaElliott MufsonNorio MiyoshiEllis S. Van EttenNoriyuki KajiElmendorf JeffreyNuno ValeElmina Mammadova-BachNunzia MontuoriEloi GaríNunzio Antonio CacciolaElvira IngleseNupur DasElzbieta Jarocka-CyrtaNuria MorralElżbieta Łodyga-ChruścińskaOctavian Tudorel OlaruElżbieta Łopieńska-BiernatOdile Poulain-GodefroyElżbieta Lorenc-KociOk Hyung NamEmanuela CollaOlaia Martinez IglesiasEmanuela FinaOlatz ZenarruzabeitiaEmanuele GiurisatoOle Valente MortensenEmanuele MauriOleg KaradutaEmanuella GrassilliOleg LunovEmiel Van Der VorstOleg MyakininEmil PaluchOleg ShuvalovEmil RudolfOlga E. SavelievaEmilia KozlowskaOlga KopachEmilia ManoleOlga KorczeniewskaEmiliano Raúl DiezOlga KruszelnickaEmmanuel RaffouxOlga Tura-CeideEnrico GarattiniOlga Y. KorolkovaEnrico MarinelliOlivier BousigesEnrico MoroOlivier NègreEnrique Gomez GomezOlivier RousselEnza SperanzaOliviero GobboEnzo LalliOmar AlhalabiEnzo Maria VingoloOmar El BounkariEric LevesqueOmer Hassan JamyEric SmithOmid AzimzadehErica GentilinOndrej VasicekErika CioneOnofrio LaselvaErika López-ArribillagaOPhir NaveErika PalmieriOren LedderErin Elizabeth SchirtzingerOrit UzielErin TaylorOsamu YoshinoErkko YlösmäkiÓscar Osorio-ConlesErmelinda MaçôasOvidiu S. StoicanErwin BrosensOxana SerovaErwin MontgomeryPablo AvanzasEssa M SaiedPanagiotis KarakaidosEsteban UrriolabeitiaPanagiotis TsagozisEstefania NuñezPandurang KolekarEster Leno-DuránPanoraia SiafakaEsther Dawen YuPaola GalluzzoEszter DuczaPaola GiordanoEtienne RousseauPaola MarcolongoEugene PonomarevPaola PiscopoEugenia BezirtzoglouPaolo AretiniEugenio GaudioPaolo Emilio PudduEugenio MontiPaolo MeneguzzoEui-Seok LeePaolo ScarpelliEuishin Edmund KimPaolo TrucilloEun Bong LeePapireddy KancharlaEun Jeong ParkParamita BasuEun-Taex OhParaskevi KarousiEunyoung TakParise AdadiEva AndreuzziPark JeongjinÉva Anna EnyedyPascal MauriceEva BagyinszkyPascal PeraldiEva KatonaPascal RowartEva PinhoPasquale MarrazzoEva RealiPasquale PiconeEva TvrdáPasqualino SirignanoEvaristo Vázquez-DomínguezPatrícia BatistaEvelise BrizolaPatricia K. CoyleEvert F.S. Van VelsenPatricia RegalEvgenia HalkiaPatrick M. PerrigueEvgeniy BolbasovPatrick SchlegelEvgeniya V. EfimovaPatrizia GuidiEvgeny ErmakovPatrizia LeoneEwa BębenekPatrizia MalaspinaEwa Gibuła-TarłowskaPaul JoyceEwa Maria KratzPaula VerissimoEwa Musiol-KrollPaulina Niedźwiedzka-RystwejEwa PiotrowskaPaulo A. GameiroEwoud B. CompeerPavel StasaFabian B. FahlbuschPavlina D. ChuntovaFabian ZanellaPawel Andrzej HermanFabien DelasprePaweł GacFabio PerrottaPaweł KleczyńskiFabio SallustioPaweł KrzyżekFabrizia BonacinaPaweł PietkiewiczFabrizio ANNIBALLIPaweł SachadynFabrizio FontanaPaweł WeichbrothFabrizio GentilePedro Carmona SáezFangcong DongPedro CasadoFangfei LiPedro De-la-TorreFarshid DayyaniPedro Perez-ManceraFarzan AkbaridoustPeer W. KämmererFarzaneh SabbaghPeggy StockFausto RigoPei-Yuan SuFawzia Bardag-GorcePengfei ZhangFederica CherchiPeng-Yuan WangFederica FogacciPeter A. SlominskyFederica FregnanPeter BališFederica Lo SardoPeter HlivakFederica LovisaPeter IgazFederica MoraniPeter KorstenFederica PapaccioPeter LittleFederica VeronesePeter MikušFederico IovinoPeter OttFederico N. SoriaPeter P. TothFederico VerdePeter PetzelbauerFelice LorussoPeter RacayFelicia Fei-Lei ChungPeter RichterFelipe Javier Chaves-MartinezPeter WestenskowFelipe ParedesPēteris TretjakovsFelipe Vadillo-OrtegaPetra PallagiFelix BroeckerPetra SurlinFelix HauschPetronella KettunenFelix Javier Jiménez-JiménezPetros P. PetrouFeng XuePetru NegreaFengzhi LiPhaneendra K. DuddempudiFerdinando Carlo SassoPhilip CharlesFerdinando PaternostroPhilippe A. GrenierFernanda Da Cruz Landim- AlvarengaPhilippe CauchieFernando C. BaltanásPhilippe GossetFernando Capela E SilvaPhilippe LewalleFernando CardonaPhilippe MarchettiFerreira Nelson FerreiraPhilippe ParolaFilip NovotnýPhilippe PourquierFilippo PieriniPia Lopez-JornetFilippo TorrisiPierfrancesco FrancoFilomena De NigrisPiergiorgio La RosaFiorella PiemontePiero RicchiutoFiras KobeissyPiero Vincenzo LippolisFlavia FontanesiPierre CordelierFlavia PauriPierre GressensFlor María Pérez-CampoPierre LebranchuFlora N. BalievaPierre Simon RöhrlichFlorence Brunet-PossentiPierre-Yves LozachFlorence LegendrePiet BorstFlorent GuérinPietro CarotenutoFlorentina Monica RadulyPinar Uysal-OnganerFlorian EnzmannPing SongFlorin OnisorPing-Hui TsengFranca CastiglionePingping HanFranca R. GueriniPingyue PanFrances Meier-GibbonsPinuccia FavianaFrancesca A. De GiorgiPio ContiFrancesca BenedettiPiotr DobrowolskiFrancesca De FalcoPiotr DuchnowskiFrancesca PeruzziPiotr G RychahouFrancesca ReggianiPiotr K. KrajewskiFrancesca Romana MariottiPiotr PoznanskiFrancesca SilvagnoPiotr SobolewskiFrancesca ZitoPirkko HärkönenFranceschelli SaraPiyush BaindaraFrancesco AsciPons-Tostivint ElvireFrancesco AsnicarPooja SabhachandaniFrancesco BennardoPoonam MudgilFrancesco CalivaPrabhu MathiyalaganFrancesco CiarleglioPrachi UmbarkarFrancesco ClapsPradeep Kumar BollaFrancesco De ChiaraPranav ShivakumarFrancesco DonatelliPranshu SahgalFrancesco FisicaroPrasanth PuthanveetilFrancesco GiudiciPrashant SharmaFrancesco MarongiuPrateek PathakFrancesco PallottiPriya MuralidharanFrancesco Paolo TambaroPriyanka DeyFrancesco RossellaPriyanka RayFrancesco StellatoPrzemysław DorożyńskiFrancesco TramaPrzemysław KołodziejFrancina Lozada NurPrzemyslaw MielczarekFrancisca DiasPu DengFrancisca Garcia-MorenoPui Wah ChoiFrancisco BustosPunit P. SethFrancisco Guillen-GrimaPushpa Raj JoshiFrancisco Lázaro-DiéguezPyrear Suhui ZhaoFrancisco RivasQaisar MaqboolFrancisco Rodrigues PintoQifei LiFranck ChiappiniQinghang MengFranck TouretQinzhe WangFranco MutinelliQiu WuFrancois MullierQiyi TangFrançois MullierRabijit DuttaFrank PfriegerRachel Bar-ShalomFrank Richard SchubertRachel NechushtaiFrank StahlRachele StefaniaFrédéric EbsteinRachid LahlilFrederic TortRadek IndraFrederik PetersRadka VaclavikovaFredrik FrejdRadomír HyšplerFu RuiRadoslav MatejFulvio LauretaniRadosław RolaGabi DrochioiuRadu AlbulescuGabriel Claudiu TenderRafael Arcángel Caparrós-GonzálezGabriel Gonzalez-EscamillaRafael BadenesGabriele NicoliniRafael BernardesGabriele ToiettaRaffaele CampisiGabriella FabbrociniRaffaele PezzilliGabriella HorváthRaffaella ComitatoGabriella IannuzzoRaghu VannamGaelle Guiraudie-CaprazRahul KumarGaetan LigatRaimondo PianaGaetano AurilioRajan Deep AdhikariGaetano PaoneRajesh BhardwajGaryfallia PeperaRajesh DurairajGatikrushna SinghRamcharan Singh AngomGaurav A. MehtaRamesh PeriasamyGaurav PandeyRamya Lakshmi RajendranGaurav V. SarodeRanganath MuniyappaGautam SethiRania AbutarboushGavin LambertRanko GacesaGelsomina MansuetoRaphaël ThuillierGema Martinez-NavarreteRappa FrancescaGema Ruiz-HurtadoRaquel Aparicio UgarrizaGen OhtsukiRasim BarutcuGen-Min LinRaul GainetdinovGentzane Sánchez-ElexpuruRaul Rodrigues-DiezGeoff ChongRavindran Caspa GokulanGeoffrey MitchellRazmik MirzayansGeorg AuburgerRǎzvan PetcaGeorge Calin DindeleganRebeca AlvariñoGeorge CarnellRebecca AichelerGeorge Edward RaingerRedi RahmaniGeorge KoliakosReece Andrew SophocleousGeorge LeondaritisRégis GuieuGeorge Mihai NitulescuReine NehméGeorge P. ParaskevasRemo CampicheGeorge PotamiasRenata ChałasGeorge TherapondosRenata MikstackaGeorge ValasoulisRenato BernardiniGeorge VartholomatosRene KoeffelGeorge Vladimirovich SharonovRené WenzlGeorges JourdiRenee KingGeorges VassauxRen-Jay SheiGeorgiana NitulescuRicardo A BattaglinoGeorgios A. ChristouRicardo CoelhoGeorgios GalaziosRicardo MagalhãesGerald CohenRiccardo BientinesiGerard M. TurinoRiccardo LombardoGerarda CappuccioRiccardo SfrisoGerd A. MüllerRichard DekhuijzenGerd BungartzRiikka MartikainenGergana DeevskaRikako SanukiGermain HonvoRikiya TakeuchiGermán GonzálezRima SlimGerman PerdomoRita PuglisiGerrit BegemannRobert E. PykeGerry LushingtonRobert GałązkowskiGesmi MilcovichRobert J. BrownGheun-Ho KimRobert K. ClemensGiada GiovanniniRobert KissGiada SebastianiRobert KleszczGianantonio SaviolaRobert KraftGianluca BaldanziRobert M. BadeauGianluca CatucciRobert NashGianluca FulgenziRobert SchmittGianluca NazzaroRobert Steven EsworthyGianluca RizzoRobert W. CoppockGianluca SferrazzaRobert ZiółkowskiGianmaria SalvioRobert-Alain ToillonGianni SoldatiRoberto Augusto Ferriz-MartínezGianpaolo PapaccioRoberto BellelliGiedrius BarauskasRoberto ColasantiGilles BreuzardRoberto PalombaGino SeravalleRoberto PivaGiordano PeriniRoberto RavazzoloGiorgia MelliRoberto RonaGiorgio MalpeliRobyn HickersonGiorgio ManginoRobyn L. PrueittGiorgio SonninoRocio I Rodriguez MaciasGiorgio TregliaRocío Ruiz LazaGiorgio TulliRocío Vila-BedmarGiovanna BorrielloRodolfo IulianoGiovanni AppendinoRodrig MarculescuGiovanni BarosiRoger GrandGiovanni CiliaRohit SrivastavaGiovanni CimminoRohollah NasiriGiovanni ColonnaRomain CapouladeGiovanni ContiRomain Maxime LariveGiovanni CrisafulliRoman S. NagovitsynGiovanni Di BernardoRomina RomanielloGiovanni N. RovielloRon KedemGiovanni PalleschiRonan LordanGiovanni PallioRonan MurphyGiovanni PaolinoRongcong LuoGiovanni RollaRosa Di LiddoGiulia BIVONARosa FontanaGiulia Di DalmaziRosa M. MartiGiulia RicciRosa María Giráldez-PérezGiulia SuaratoRosa PurgatorioGiuliano BernalRosa SacedónGiuseppe BrisindaRosa VonoGiuseppe CirilloRosalba SiracusaGiuseppe D. CiccotostoRosanna Di PaolaGiuseppe GattusoRosario CaltabianoGiuseppe LanzaRosario DonatoGiuseppe MurdacaRosario SerpicoGiuseppe MusumeciRoss SummerGiuseppe PizzolantiRossella MieleGiuseppe PortellaRouf BandayGiuseppe ReimondoRoxana RacoviceanuGiuseppe RivaRoy DrissenGiuseppe SantarpinoRubén CiriaGiuseppe SciamannaRuben MedinaGiuseppina CatanzaroRudi BeyaertGloria Álvarez-SolaRudolf HergesheimerGloria PerazzoliRudolf KieferGobinath ShanmugamRui SongGolara GolbaghiRuifeng HuGomes CátiaRupendra ShresthaGonzalo HaroRusso EleonoraGourisankar GhoshRuth PrasslGraham F BradyRuwei LinGrasiele SausenRwik SenGrażyna Odrowaz-SypniewskaRyoichi AsakaGreeshma ThrivikramanRyoma MichishitaGregor NorcicRyou TanakaGregory J LandryRyousuke MatsudaGrigoris AmoutziasRyszard PlutaGrzegorz GrześkRyuji HamamotoGrzegorz KreinerSabata MartinoGrzegorz TrybekSabbir KhanGrzegorz ZagułaSabina SevcikovaGuangde JiangSabine BrandtGuangfu WuSabine WipperGuangxu MaSabrina CorbettaGuannan WangSabrina ReinehrGuennadi SaikoSadanori AkitaGuglielmina ChimientiSaeid GhavamiGuillaume BastiatSafaa KaderGuillaume BlanchetSagar ChittoriGuillaume E. CourtoySahib ZadaGuillaume GiraudSahil SharmaGuiting LinSaikat BalaGulam RabbaniSaima ImranGun Woo LeeSajjad AhmadGundula Gesine Schulze-TanzilSajjad ShiraziGunnar BoysenSalah AmashehGuoqing QianSalina GairheGuozheng WangSalma KaocharGurdeep MarwarhaSalman IslamGuruprakash SubbiahdossSalman M. HyderGustavo Alberto ChiabrandoSalvatore CocuzzaGustavo YannarelliSalvatore FeoGuy G. PoirierSaman WarnakulasuriyaGuy LadamSamantha DonsanteGuy RostokerSambuddha BasuGuzzo GabriellaSamir BarghoutGwang Hyeon EomSamir ČimićGyorgy KerekesSamit ChakrabartyGyörgy NádasySamuel D. RobinsonGyörgy Tibor BaloghSamuel E. AdunyahHabil. Agnieszka Dettlaff-PokoraSamuel X. ShiHae June LeeSandeep Kumar BarodiaHaifei ShiSandesh PanthiHalina JurkowskaSandipan DattaHamed MontazeriSandra DonniniHan Naung TunSandra LejnieceHana StarobovaSandra SigalaHankyu LeeSang-Han LeeHan-Mou TsaiSangu MuthurajuHanna KmitaSanjeev ChoudharyHann-Chorng KuoSanket MishraHans Christian BeckSantiago Garcia-VallveHans-Gert BernsteinSantiago NavarroHarald KolmarSantosh GhoshHarald MischakSantosh PandaHardy RideoutSara BernardiHarleen KaurSara E. DanziHarm HaakSara MissagliaHarriet GroomSara ScuteraHarry BjörkbackaSarah L. KeaseyHarsharan S. BhatiaSarah SerticHaruki KoikeSarel HalachmiHaruki UsudaSaša FrankHatice KumruSatish Balasaheb NimseHecson Jesser SegatSatomi OnoueHeidi NeubauerSatoru KaseHeiko WeydSatoru KobayashiHeinz H. CoenenSatu HepojokiHelen FillmoreSaudade AndréHelena FelgueirasSawsan A. ZaitoneHelmy MokhtarScott McCombHemant K. MishraSe Jin ParkHenri GruffatSean ChiaHenrik NielsenSean P. DidionHenry P. GodfreySebastian FerriHenry S. ParkSebastian GranicaHenry SutantoSebastian SjöqvistHerbert Ryan MariniSebastiano CiccoHerman E. PopeijusSebastiano TorrisiHerwig CerwenkaSébastien PenninckxHesam BabahosseiniSebastjan BevcHidehiko KikuchiSeigo OhbaHideki FujiiSeil KimHideki SugiiSekiya TakashiHidemasa BonoSelcuk KucukseymenHimangshu SonowalSemih Can AkıncılarHimanshu KathuriaSemyon V. DudkinHimavanth Reddy GatlaSenthil ChinnakannanHiroaki KonishiSeockhui KangHiromu KondoSeong Soo A. AnHiroshi FukushimaSerena BianchiHiroshi HandaSerena LucottiHiroshi IshiiSerena VeschiHiroshi KajiyamaSerge WeisHiroshi KitohSergei G. GaidinHiroshi MurakamiSergei Nikolaevich ShchelkunovHiroshi TsutsumiSergey A. SinenkoHiroshi YoshitakeSergey G. KhasabovHirotomo KatoSergey KolomeǐchukHiroyasu SakaiSergey KozlovHiroyuki UetakeSergey MalkinHolger A. LindnerSergey SedykhHolger ErbSerumJi-Hun KangHongda CaoSeth TarrantHongjun WangSeung Hee LeeHongnan CaoSeung Hwan LeeHoon KimSeung-Gu YeoHorea FeierSevdalina LambovaHornyák IstvánSeverin RodlerHorst Von BernuthShahin HomaeigoharHossam AshourShahrokh GhobadlooHossein HosseinkhaniShailesh K. ShahiHsiao Tien LiuShalaka MulherkarHsiao-Hsuan KuoShambhabi ChatterjeeHsin-Yuan ChenShane ThomasHsueh-Wei ChangShankar Jaikishan EvaniHuafeng WangShao-Wen HungHubert SchorleSharmila FagooneeHugues Allard-ChamardSharzad EmamikiaHugues JacobsShashank PandeyHui WangShashank SrivastavaHuibin LvShay Ben-ShacharHui-Ching ChuangShazia BanoHui-Chun HuangSheikh RiazuddinHui-Hua HsiaoSheila DonnellyHui-min WangShekhar SahaHui-Wen ChiuShekoufeh AlmasiHung Huey-ShanSheng-Chieh LinHung-Chi ChengShenghong HeHung-Lung ChiangSheng-Sheng YuHung-Pin TuShiang-Jong TzengHung-Yi ChuangShian-Ren LinHung-Yin LinShi-Bei WuHung-Yu LinShigeki SugawaraHussein KaddourShih-bin SuHwajin KimShih-Hsien HsuHwa-Jung KimShiho OhnishiHyemin KimShihori TanabeHyeong-Geug KimShih-Yen LoHyeonji KimShikha SainiHye-ra LeeShilpa ThakurHyesun KimShin EnosawaHyun Joo AnShin HamadaHyunju LeeShin Ichi NurekiHyun-Woo ParkShin KashimaIbrahim KassisShin NishioI-Chen PengShing-Hwa LiuIda AringerShintaro FujiharaIftekhar MahmoodShintaro NakanoIgnacio BenedictoShiro SuetsuguIgnacio VerdeShiv BharadwajIgor DumicShizuka UchidaIgor Moiseyevich KvetnoySho TsukamotoIgor PrudovskyShobini JayaramanIgor SlivacShofiul AzamIl Soo MoonShoichi FujiiIlaria BortoneShravan MorlaIlaria CampesiShrikant PawarIlaria TonazziniShuchi WangIldiko SzantoShu-Jui KuoIleana ManduteanuShulin QinIlenia CalcaterraShungo EndoIlkka PaateroShu-Pin HuangIman MohammadzadehShuvashis DeyImmacolata PietraforteShyam Kumar GudeyImran HouseSiddharth ShuklaImran NooraniSieghart SopperIna RudloffSigrun EickInes MármolSi-Han WuIng-Ming ChiuSilva BortolussiInken WohlersSilvana AlfeiInna SekirovSilvia BrunelliIoanna NikitopoulouSilvia Di AgostinoIoannis GiantsisSilvia DragoniIoannis KoutelidakisSílvia J. BidarraIoannis KyrgiosSilvia Jiménez-MoralesIoannis MorianosSilvia LiskovaIoannis Ntanasis-StathopoulosSilvia MarracciIolanda LázaroSilvia MonteiroIon PetreSilvia PasquiniIra PastanSílvia Pérez-LluchIrena Baranowska-BosiackaSilvia RocchiccioliIrena IlicSilvia SvegliatiIrena NalepaSilvia VivarelliIrene LidorikiSilvio MaringhiniIrene MadrigalSimon L. GoodmanIrene RosaSimona Delle MonacheIrfan A. RatherSimona LattanziIrina KondakovaSimona Lucia BavaroIrina MateiSimona MitevaIrina S. MoreiraSimona NeriIris Maria ForteSimona SacchiniIrshad SharafutdinovSimona SalernoIsaac CanalsSimone GiannecchiniIsabel CorreiaSimone GrassiIsabel LegazSimone PatergnaniIsabella ZironiSimone VanoniIsidro MachadoŠimons ŠvirskisIsrael Carreira-BarralSina NaserianIstván SzokodiSiroos MirzaeiI-Ta LeeSiva Krishna AngalakurthiI-Tsang ChiangSivaraman NatarajanItziar Lamiquiz MoneoSjoerd T. T. SchettersIvan BarvikSjors G.J.G. In’t VeldIvan ChenchevSmitha SripathyIvan SalazarSnježana Mardešić-BrakusIvan V. NechepurenkoSoichiro YamamuraIvan Y. IourovSon Long HoIvana GrgićSoňa ČiernikováIvana JochmanováSoňa LegartováIvana KholováSonali NashineIvana RumoraSónia C. CorreiaIvana SamarzijaSonia NathIvana ŠkrlecSoo-ah KimIvaylo DimitrovSøren ChristensenIvonne NelSoumya PoddarIwona BryndalSourav RoyIwona KucybałaSowmya SrinivasanIwona RybakowskaSpyridon KintziosIwona WertelSriharsa PradhanIzabela Małysz-CymborskaSrinivas NellimarlaIzabela ZakrockaSriram SundaravelJ. Catharina DuvigneauStamatis GregoriouJ. Robert HoggStanescu Ana Maria AlexandraJ. Sven D. MieogStavroula IliaJacek C SzepietowskiStefan Cristian VesaJacek DrobnikStefan HeberJacek KujawskiStefan MihaicutaJacek KwiecienStefan NierkensJacek NowaczykStefan ZielenJacek R. WilczyńskiStefan ZóradJacopo LucchettiStefan-Adrian StrungaruJacopo SabbatinelliStefania CantoreJacqueline CloosStefania CoccoJacques Le PenduStefania CrociJacques TurgeonStefania GalimbertiJacques ZimmerStefania LamponiJade LoggerStefania MarcuzzoJae Ho LeeStefania MerighiJae Hoon BahnStefania MitolaJae Min ChoStefania ScaliseJae Myoung NohStefanie MaurerJakir HossainStefano BellostaJakub Dalibor RybkaStefano CiardulloJakub Kła̧czStefano DastoliJakub MorzeStefano FornasaroJakub RokStefano Giuseppe CaraffiJakub SuchodolskiStefano GrolliJames A. MarrsStefano LuinJames F. MorleyStefano P. PiottoJames K. RussellStefano PalermiJames O'CallaghanStefano RapiJames P. BennettStefano RealeJames R. S. HockerStefano SotgiuJamoliddin RazzokovSteffen PockesJan KrijtStepan KutilekJan LakotaStephan Joel ReshkinJan MejzlikStephan LindemannJan NovákStephane BourqueJan SperlStéphane ChavanasJan StępniakStéphane PoitevinJan ZivnyStephanie BaudJana LipkovaStéphanie BlockhuysJana MašlankováStephanie DauthJanaki IyerStephanie HehlgansJane Kasten-JollyStephanie S Tseng-RogenskiJanet R. MorrowStephen A. MorrisJanice M. PluthStergios BoussiosJanin HenkelSteve GameiroJanja TrčekSteve LeuJános G. FilepSteven M. PascalJanusz BlasiakSteven Paul NisticòJared BarrottSteven StackerJari LouhelainenSubhadip ChoudhuriJaris ValenciaSubramanian VenkatesanJaromir AstlSudha SharmaJaroslav A HubacekSudhanshu RaikwarJarosław CzyżSudip MondalJarosław J. SakSudip PaulJar-Yi HoSUGANUMA KeisukeJasenka Gajdoš KljusurićSuk Ling MaJasmina VidicSuman ChowdhuryJasmine SinhaSuman DuttaJasminka ŠtefuljSuman MukhopadhyayJasna MetovicSumit MukherjeeJason L. BurkheadSumit MurabJavier Avila-RománSundaresan GobalakrishnanJavier Molina-CerrilloSuneet KaurJavier SantabarbaraSung Hwan KiJaw-Yuan WangSung Noh HongJay E. JohnsonSung Soo AhnJaya Prakash GollaSung-Hsin KuoJayaum S. BoothSung-Kyo KimJaycob D. WarfelSungSoon FangJayne MurraySunil KumarJean AmiralSunil SirohiJean GuibourdencheSupawadee SuksereeJean McBryanSupreet AgarwalJean MukherjeeSupriya RavichandranJeanette A.M MaierSurendra RajpurohitJeanine M. WalengaSuzanne Faure-DupuyJean-Luc PoyetSven FlemmingJean-Marie RuysschaertSvetlana GalkinaJean-Pierre GagnerSy Duong-QuyJeff SandsSyed Haris OmarJeffrey E. IndesSyed Husain Mustafa RizviJeffrey J. AdamoviczSyed MehdiJelena Korac PrlicSyed Sayeed AhmadJelena MuncanTae Sup LeeJelena ViderTae-hoon ChungJenchih TsengTai-long PanJennifer CaseyTaishi HataJennifer HerrmannTakaaki HirotsuJeonghwan GwakTakao ShimazoeJeong-yeon LeeTakashi KuritaJer-An LinTakashi ObamaJer-Ming ChangTakayuki KatayamaJerónimo GonzálezTakayuki OkamotoJerzy BeltowskiTakemi OtsukiJerzy ChudekTakemichi FukasawaJerzy JaroszewiczTakenobu KatagiriJerzy-Roch NoferTamás Kovács-ÖllerJessica BauerȚarălungă Dragoș DanielJessica HoffmanTatiana ScorzaJessica Maria AbbateTatsuhiro HisatsuneJessica MwinyiTatsuo KandaJessica T. LeonardTatsuya MorimotoJesus M. Martin-CamposTatsuya ShirahataJesus Perez-LosadaTaxiarchis KatsinelosJi Hyeon AhnTeayoun KimJia (Joe) Long ZhuoTeikichi IkuraJia Ping WuTetsufumi ItoJiabing FanTheodora PapamitsouJiacheng SunThierno Madjou BahJian ShiThomas CluzeauJianfeng HuangTing-yi LinJiang-Yun LuoTokuko HaraguchiJianjun WenTomas BertokJianmin SuTomasz StompórJianming QiuTomoaki MiyagawaJianming WuTomohiro MizobataJianxuan WuTomohisa BabaJie GaoToos DaemenJie WangTorchio AlessandroJi-Hyeon OhToshio HattoriJill MorganToyoaki OhbuchiJin Hee KimTriantafyllos DidangelosJin ZhangTruong Phuoc NghiaJin'An WangTsung-I HsuJing ChenTsung-Lin ChengJing WangTsuyoshi FukushimaJing ZhangTsuyoshi KimuraJinghua WangTsuyoshi OkuraJingquan WangTuo MengJingsong ZhouTzipora S. Karin Eisinger-MathasonJinn-Rung KuoUrsula SandauJinwei ZhangValentina Oana BudaJiongwei WangValentina OliveriJiří MoláčekValeri V. MossineJiro KishimotoVasileiou PanagiotisJitendra KanaujiyaVasily A. PopkovJi-Yeong BaeVasily KashtalapJi-Yih ChenVenkata P. MantripragadaJ.L. Gómez ArizaVeronika KarcagiJo CaersVijay AvinJoachim SchmittVijay Sagar MadamsettyJoachim StorsbergViktor V. RevinJoan C. KrepinskyVinayak JoshiJoan Carles Escola-GilVincenza GianfrediJoan OlivaVincenzo NicolettiJoana BickerVladimir DyakonovJoana CaetanoVladimir GureevJoana FangueiroVladimir PustylnyakJoanna FilipowskaW.D. GrimmJoanna WróblewskaWei LiJoanna Zawacka-PankauWei Long NgJoanna ZemłaWei-Shiung LianJoanne Van RynWen Chieh LiaoJoão H. P. M. SantosWen-Chiuan TsaiJoão M. C. TeixeiraWen-Fu LaiJoão M. SantosWen-Lung MaJoão Miguel Marques Dos SantosWen-Wei ChangJoaquim CarrerasWładysław LasońJoaquim C.G. Esteves Da SilvaWojciech KalasJoaquim OristrellWoon-Man KungJoaquín María Campos RosaWu-Chou SuJochen PölingXiaoyan ChenJochen SchneiderXiaoyu LiJohann SellnerXijun PiaoJohanna RommensXinbo LiJohn Bertram VincentXinwei LiJohn EllulXuqiao ChenJohn H. PyneYajuan SuJohn HortonYaozong YeJohn KershnerYash RavalJohn ManionYasuhiro KosugeJohn McGinnissYasumasa OkazakiJohn O. OnukwuforYasuteru ShigetaJohn T. BatesYau Kei ChanJohura AnsaryYi Hui WuJon FordYi-Chou HouJon P. GoldingYin-Hwa ShihJonas BucevičiusYoichi MorofujiJonathan GrasmanYoichi SakuradaJonathan O’Keeffe AhernYong Il HwangJonathan ReichnerYongjun SuiJonathan SantoroYoshifumi SaijoJong Hyuk SungYoshihiro FukumotoJong Kook ParkYoshikazu KitanoJongseong KimYoshinobu KariyaJong-Sup BaeYoshinori ArisakaJoon W. ShimYoshinori NakazawaJoonghoon ParkYoshiro HoraiJoost Van Der VorstYousuke IshitsukaJordi Martorell-MarugánYue QiJörg HeroldYu-Hsiang KuanJörg KleeffYuichiro FujiedaJörg LindenmannYu-Jen ChenJorge A. MoralesYuji HirowatariJorge AntunesYuji NadataniJorge Sastre-SerraYuji ShimizuJosé A. CañasYu-Jung ChengJosé Ángel ArmengolYuki AkagiJose Antonio Vega AlvarezYu-Ling LinJosé Avendaño-OrtizYuliya I. RaginoJosé Bernardo Noronha MatosYun-Bo ShiJose Luis LavanderaZdenka MuchováJosé Luis ZugazaZehuan LiaoJose M. GarcíaZhaohai QinJosé M Rodrigo-MuñozZhenglin GuJosé Manuel Pérez De La LastraZhipeng LiuJosè Manuel PionerZihao OuJose Manuel Rodriguez-VargasZiwen WangJosé R. PinedaZoltán HeroldJosé Rafael De AlmeidaZong Sheng GuoJosef FinstererZsolt GállJosef HalamekZygmunt Warzecha


